# Pulmonary *Actinomyces graevenitzii* Infection: Case Report and Review of the Literature

**DOI:** 10.3389/fmed.2022.916817

**Published:** 2022-06-10

**Authors:** Yuan Yuan, Ziliang Hou, Dan Peng, Zhenchuan Xing, Jinxiang Wang, Shuai Zhang

**Affiliations:** Department of Pulmonary and Critical Care Medicine, Beijing Luhe Hospital, Capital Medical University, Beijing, China

**Keywords:** pulmonary actinomycosis (PA), *Actinomyces graevenitzii*, bronchoscopy, matrix-assisted laser desorption/ionization time-of-flight mass spectrometry, consolidation

## Abstract

**Background:**

Pulmonary actinomycosis (PA), a chronic indolent infection, is a diagnostic challenge. *Actinomyces graevenitzii* is a relatively rare Actinomyces species isolated from various clinical samples.

**Case Presentation:**

A 47-year-old patient presented with a 3-month history of mucopurulent expectoration and dyspnea and a 3-day history of fever up to 39.0°C. He had dental caries and a history of alcoholism. Computed tomography (CT) images of the chest revealed a consolidation shadow in the right upper and middle lobes, with necrosis containing foci of air. *Actinomyces graevenitzii* was isolated from bronchoalveolar lavage fluid (BALF) culture and was identified by matrix-assisted laser desorption/ionization time-of-flight mass spectrometry. He received treatment with intravenous piperacillin-sulbactam for 10 days and oral amoxicillin-clavulanate for 7 months. His clinical condition had considerably improved. The consolidation shadow was gradually absorbed.

**Conclusion:**

Early diagnosis and treatment of pulmonary actinomycosis are crucial. Bronchoscopy plays a key role in the diagnostic process, and matrix-assisted laser desorption/ionization time-of-flight mass spectrometry (MALDI-TOF/MS) is an accurate tool for *Actinomyces* identification.

## Introduction

Actinomycosis is a rare chronic disease caused by the anaerobic Gram-positive bacteria *Actinomyces spp*, which normally inhabits the human oral cavity, digestive tract and genital tract (1). Due to its non-specific clinical manifestations and imaging characteristics, actinomycosis is easily misdiagnosed or missed. Pulmonary *Actinomyces graevenitzii* infection is extremely rare. The present case is the only case of pulmonary actinomycosis in our department. In this report, we describe it in detail and review the pertinent literature to improve our understanding of this disease, avoid misdiagnosis, and provide evidence for its clinical diagnosis, treatment, and prognosis.

## Case Presentation

A 47-year-old man was admitted to the Department of Pulmonary and Critical Care Medicine, Beijing Luhe Hospital, Capital Medical University (Beijing, China) with a 3-month history of mucopurulent expectoration and dyspnea. He was initially diagnosed with bronchitis at a health center in the local town and did not undergo chest radiography and laboratory tests. He received intravenous antibiotics for 14 days, but his symptoms were not relieved. Three days before admission, he started having a fever with a maximum temperature of 39.0°C. He did not report chest pain, hemoptysis, headache, vomiting, and other symptoms. His past medical history was unremarkable with no exposure to chronic diseases, infectious diseases or allergy. The patient was a worker and lived in an urban setting. No data on major epidemic, family history, toxic habits, or occupational exposure was reported. He was married and denied any sexually transmitted infections or drug abuse. He had a 30-year smoking history (20 cigarettes per day on average) and a 20-year history of alcohol intake of a 150 mL daily.

At admission, the patient was febrile with a temperature of 38.9°C, tachycardiac (107 beats/min), and tachypneic (22 breaths/min). His blood pressure (110/80 mmHg) and oxygen saturation (99%, measured by pulse oximetry while breathing ambient air) were normal. Examination of the oral cavity showed poor hygiene. There were wet rales in the right lung. The auscultation of the heart revealed regular rhythm without murmur. The abdomen was tender, and there was no organomegaly. No abnormal findings were found in the neurologic examination.

**FIGURE 1:**
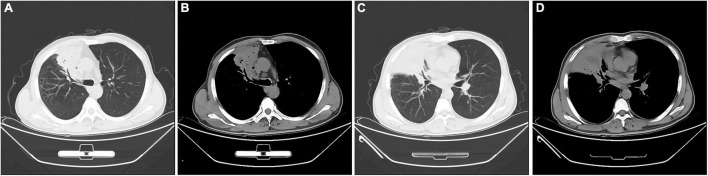
Chest computed tomography images at admission. A consolidation shadow in the right upper and middle lobes, with necrosis containing foci of air. **(A,C)** lung window; **(B,D)** mediastinal window.

The laboratory data was as follows: the peripheral blood white cell count was 8,740 cells/μl with 78.40% neutrophils, and 14.10% lymphocytes, and 6.70% monocytes; hemoglobin 110 g/L, the hematocrit 34%, the platelet count 447,000 per cubic millimeter. The C-reactive protein (71.67 mg/L), and erythrocyte sedimentation rate (100 mm/h) were significantly elevated. The PCT was mildly increased (0.13 ng/ml). Biochemical analysis of liver and renal showed normal range except slightly decreased albumin (32.1 g/L). Coagulation profile was normal and the serum tumor markers were negative. The results for possible connective tissue disease, such as antinuclear antibody, extractable nuclear antigen, and antineutrophil cytoplasmic antibody, were also negative. The serum IgM antibodies specific for respiratory pathogens, including Legionella pneumophila antibody, Mycoplasma pneumoniae antibody, Chlamydia pneumoniae antibody, influenza A virus antibody, and influenza B virus antibody, revealed no abnormality. The serum (1,3)-beta-D-glucan test (G test), the galactomannan test (GM test), and the serum TB test (T-SPOT) were all negative. The results of two blood cultures were negative. No pathological findings were detected in repeated sputum smears and cultures. A chest computed tomography (CT) scan showed a consolidation shadow in the upper and middle lobes of right lung with foci of necrosis (Figure 1).

**FIGURE 2:**
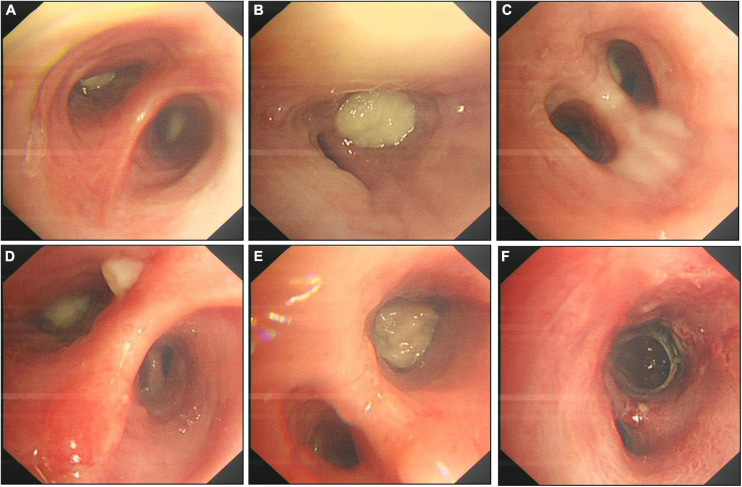
A series of bronchoscopy images. The first bronchoscopy images **(A–C)**, the secondary bronchoscopy images **(D–F)**. **(A)** The medial and lateral segments of the RML were blocked by purulent yellow secretions. **(B)** The medial subsegment of the RML was completely obstructed by an endobronchial white necrotized mass. **(C)** The media subsegment of the RML became unobstructed after suction. **(D)** The medial and lateral segments of the RML were blocked by purulent yellow secretions. **(E)** The medial subsegment of the RML was completely obstructed by a an endobronchial white necrotized mass. **(F)** The media subsegment of the RML became unobstructed after suction. RML: Right middle lobe.

**FIGURE 3:**
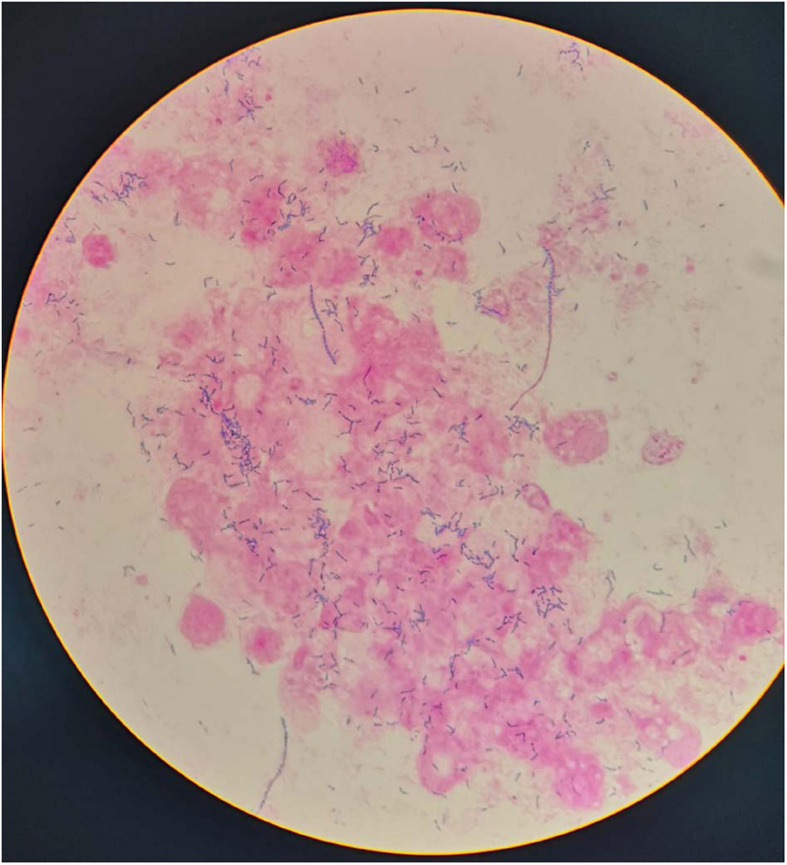
Gram stain of the bronchoalveolar lavage fluid.

**FIGURE 4:**
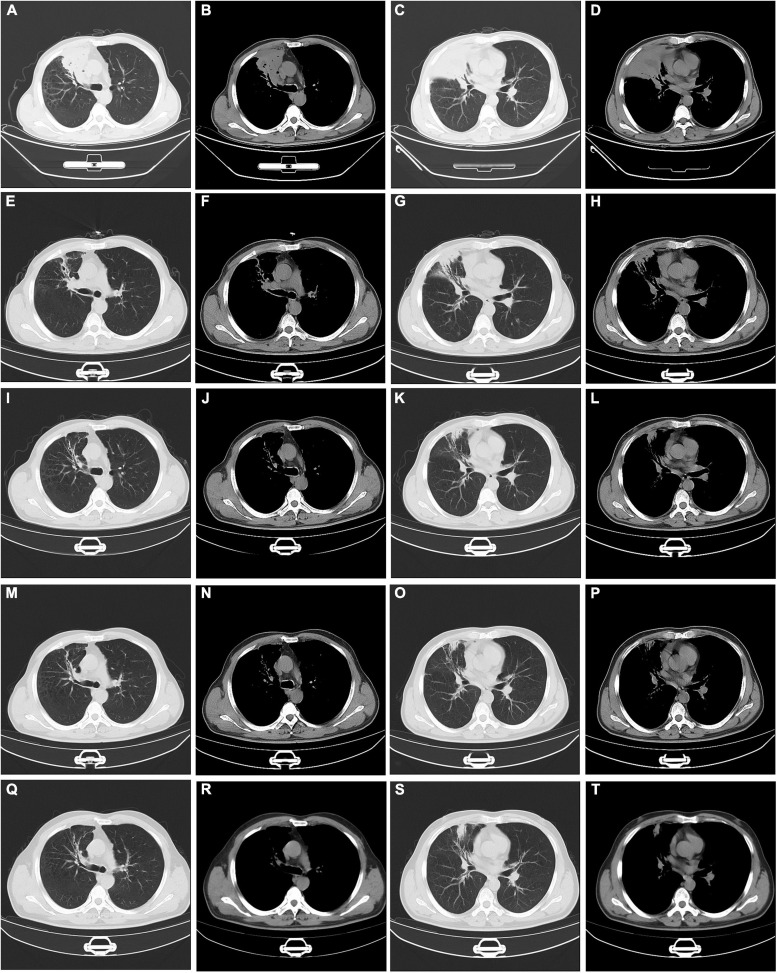
Serial changes on chest computed tomography findings. Chest CT at discharge **(A–D)**. Chest CT at one month’s follow-up **(E–H)**. Chest CT at three months’ follow-up **(I–L)**. Chest CT at five months’ follow-up **(M–P)**. Chest CT at seven months’ follow-up **(Q–T)**. CT: computed tomography.

**FIGURE 5:**
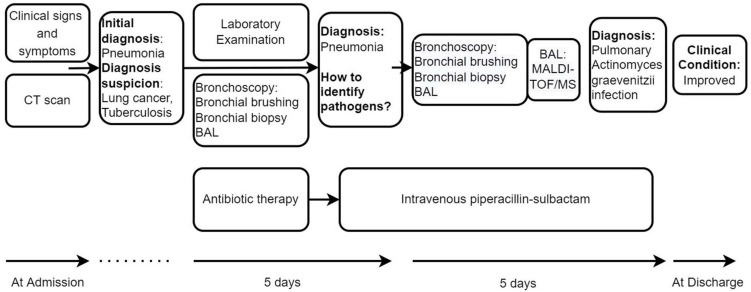
Timeline with the most relevant data of the clinical case.

Initially, We considered pneumonia as a possible diagnosis, which needed to be identified with lung cancer or pulmonary tuberculosis, and Antibiotic therapy with intravenous piperacillin-sulbactam was initiated. To make a definitive diagnosis, we performed a flexible bronchoscopy (Figures 2A–C), revealing purulent secretions in the medial and lateral segments of the right middle lobe (RML), drastically obstructing the lumen. After suction, the purulent secretions of the medial segment were reduced and the lateral segment was unobstructed. Then, bronchial brushing (BB), bronchial biopsy, and bronchoalveolar lavage were performed in the medial segment of the RML. Bronchial mucosal biopsy and BB specimens revealed only non-specific inflammation of the mucosa, and no pathogens were observed in the bronchoalveolar lavage fluid (BALF) through routine specimen smear and culture. On the third day after admission, the patient had no fever and continued to receive intravenous piperacillin-sulbactam therapy. To reduce secretions and search pathogens, we performed a second flexible bronchoscopy (Figures 2D–F), showing purulent secretions in the medial segment of the RML and the anterior segment of the right upper lobe (RUL). The BALF sample of the second time was sent for microbiological analysis. We took an appropriate amount of BALF samples for Gram staining, which is blue-purple (Figure 3). The BALF specimen was vortexed and shaken for 30–60 s. We used a sterile tool to spread a 10 μL calibration sample on the surface of the blood plate. The inoculated blood plate was incubated at 35°C for 48 h in a carbon dioxide incubator (5–10% CO2). The colony growing on the culture medium was smeared on a target plate. After drying, it was covered using a 1 μL Bruker matrix solution. When it was dry, the target plate was loaded into the machine: Microflex LT (Bruker Daltonics, Bremen, Germany). Matrix-assisted laser desorption/ionization time-of-flight mass spectrometry (MALDI-TOF/MS) for the identification of pathogens was performed, and *Actinomyces graevenitzii* was identified from the BALF. These results indicated that the pulmonary lesion of our patient was caused by *A. graevenitzii*. After administration of antibiotic treatment with piperacillin-sulbactam for ten days, the clinical condition of the patient improved obviously, and the CRP dropped to 29.34 mg/L, though the pulmonary lesions on the chest CT scan (Figures 4A–D) were not significantly absorbed. The patient was discharged with oral amoxicillin-clavulanate. A timeline with all relevant data from this clinical case is available in Figure 5. At the 7-month follow-up, the clinical condition of the patient was getting better and the chest CT scans (Figure 4) revealed that the consolidation shadow of the right lung was gradually absorbed.

## Discussion

Nowdays, more and more pulmonary actinomycosis is identified, through its clinical manifestations and imaging characteristics lack specificity. However, to date, there are only few reports of pulmonary *Actinomyces graevenitzii* infection. In the present study, we described a case of pulmonary *Actinomyces graevenitzii* infection diagnosed by microbiological identification through matrix-assisted laser desorption/ionization time-of-flight mass spectrometry (MALDI-TOF/MS). After treatment with targeted antibiotic, the clinical manifestations and imaging presentations of the patient gradually improved.

Actinomycosis is a slowly progressing granulomatous disease caused by *Actinomyces* species. *Actinomyces* is an anaerobic gram-positive bacterium, belonging to the human commensal flora of the oropharynx, gastrointestinal tract, and urogenital tract. Some species of *Actinomyces* have already been described, including *Actinomyces israelii* (2, 3), *Actinomyces odontolyticus* (4), *Actinomyces viscocus* (5), *Actinomyces meyeri* (6), *Actinomyces gerencseriae* (7), *Actinomyces naeslundii* (8), and *Actinomyces graevenitzii* (9–16). Among them, *A. israelii* is the most prevalent species isolated in human infections (9, 17–19). However, in our case, *A. graevenitzii* was identified as the pathogenic bacteria isolated from bronchoalveolar lavage fluid. *A. graevenitzii* was first described in 1997 by Ramos et al. (20) in human clinical specimens (three respiratory and one bone samples). Being a catalase-negative, facultatively anaerobic, gram-positive, rod-shaped organism, *A. graevenitzii* is isolated almost exclusively from oral or respiratory sites and may have a unique ability to cause clinical actinomycosis (21). Although the clinical prevalence and pathogenic potential of *A. graevenitzii* is little known, the frequency of isolation from clinical specimens is increasing. We performed a PubMed search with the term “*Actinomyces graevenitzii*” or “pulmonary *Actinomyces graevenitzii* infection,” and found that seven case reports in patients with pulmonary *Actinomyces graevenitzii* infection had been published (1, 6, 10–15). We reviewed the eight cases involving pulmonary actinomycosis in patients with *A. graevenitzii* infection, including our patient. The clinical features of the cases are shown in Table 1.

**TABLE 1:** The characteristics of the eight cases of pulmonary *Actinomyces* graevenitzii infection.

Case	Year	Age	Symptom	Comorbidity	Diagnoses initially	Chest CT Finding	Invasive	Confirmatory	Identification	Treatment	Treatment	Outcome
No		/Sex			Suspected		Examination	Specimen	Methods		Duration	
1	2022	47/M	Cough, dyspnea	Smoking history	Bacterial pneumonia	A consolidation shadow	Bronchoscopy	BALF	MALDI-TOF MS	PIP-SBT	10 days	Improved
			For 3 months;	Alcohol history	TB	*Trans*-fissural extension				IV		
			Fever for 3 days	Dental caries	Lung cancer	With necrosis				AMC PO	7 months	
2 (15)	2018	75/M	Low-grade fever,	GBS	Lung cancer	A nodule with a cavity	Bronchoscopy	BALF	MALDI-TOF MS	SAM IV	1 week	Improved
			dry cough	Periodontitis	AFB infection	In the right upper lobe	EBUS-GS		PCR amplification	AM IV	1 week	
			For 10 days	Smoking history	Actinomycosis				16S rRNA sequencing	AM PO	2 months	
3 (14)	2017	58/F	Fever	DVT	NR	Bilateral hilar, mediastinal	Bronchoscopy	Lymph node	NR	AMC	NR	Improved
			For 6–8 weeks;	TR		Lymphadenopathies,	EBUS-TBNA	biopsy		and CC		
			Cough, dyspnea,	Bronchial asthma		Alveolar infiltrate						
			Loss of appetite,			In the right lower lobe						
4 (13)	2014	35/M	Cough for 1 year;	Travel to Sicily,	TB	A nodule with cavitation	Bronchoscopy	BALF	NR	AMX	6 weeks	Improved
			Night sweats,	Italy		In the right middle lobe						
			Cough for 1 week									
5 (12)	2012	38/F	Fever,	Visit Los Angeles,	Metastatic tumors	Multiple round lesions	Bronchoscopy	BALF	PCR amplification	AMX	2 months	Improved
			Dry cough	California	Coccidioidomycosis	Located on both lobes,	EBUS-GS		16S rRNA sequencing			
			For 8 days	For 3 days		With partial cavity formation	VATS					
6 (11)	2012	69/M	Low-grade fever	Smoking history	CAP	Multiple consolidation	Bronchoscopy	Lung biopsy	PCR amplification	AM IV	1 month	Improved
			Night sweats		Malignancy	With air bronchograms	VATS		16S rRNA sequencing	AM and	6 months	
			For 2 months			In the lungs bilaterally				CLR PO		
7 (16)	2007	52/M	Fever, cough	CD for 9 years	TB	Diffuse patchy consolidation,	Bronchoscopy	BALF	NR	PEN and	5 weeks	Improved
			Night sweats	Infliximab	Bacterial pneumonia	Ground-glass opacities,				CLR PO		
			For 12 days	IS medication	Atypical pneumonia	Branching centrilobular				DOX PO	NR	
				Exposure to TB		Nodular opacities						
8 (10)	2005	46/M	Fever, cough,	CAD	CAP	A right-upper-lobe cavity	Needle	Sputum	PCR amplification	AM PO	6 months	Improved
			Night sweats,	Hypertension	TB		Aspiration		16S rRNA sequencing			
			Weight loss	CHF	Actinomycosis							
			For 4 weeks	Drug use history								

*M, Male; F, Female; GBS, Guillain-Barre syndrome; DVT, Deep vein thrombosis; TR, Tricuspid regurgitation; CD, Crohn’s disease; IS, Immunosuppressive; TB, Tuberculosis; CAD, Coronary artery disease; CHF, chronic congestive heart failure; AFB, Acid-fast bacillus; CAP, Community-acquired pneumonia; EBUS-GS, Endobronchial ultrasonography with a guide sheath; EBUS-TBNA, Endobronchial ultrasonography-transbronchial needle aspiration; VATS, Video-assisted thoracoscopic surgery; BALF, Bronchoalveolar lavage fluid; MALDI-TOF MS, Matrix-assisted laser desorption/ionization time-of-flight mass spectrometry; PCR, Polymerase chain reaction; PIP-SBT, piperacillin-sulbactam; AMC, amoxicillin-clavulanate; SAM, Ampicillin-sulbactam; AM, Ampicillin; AMX, Amoxicillin; CC, Clindamycin; CLR, Clarithromycin; PEN, Penicillin; DOX, Doxycycline; IV, Intravenously; PO, orally; NR, Not reported.*

The pathogenesis of actinomycosis remains unclear, however some factors probably promote the disease (1). Pulmonary actinomycosis results mainly from aspiration of oropharyngeal or gastrointestinal secretions, and poor oral hygiene (22), pre-existing dental disease (4, 23), and alcohol abuse are the important predisposing factors for actinomycosis. Other risk factors include chronic lung diseases, such as emphysema, chronic bronchitis, and bronchiectasis (1). When local mucosal tissue is damaged, infection occurs in the form of continuous growth through the anatomical barrier, leading to the formation of abscesses and fistulas (17). In the eight cases, our case had dental caries, one case had periodontitis (15), and another case had septic mouth with several teeth missing (14). In our case, the patient also had a drinking habit. Actinomycosis is frequently associated with immunocompromised states. Cohen R D et al. described a case of pulmonary actinomycosis in association with infliximab treatment for Crohn’s disease (16). One case of disseminated coinfection with *A. graevenitzii* and Mycobacterium tuberculosis has been reported (10). Besides those, it can also affect healthy people (4). S Gliga et al. reported a healthy young man of pulmonary *A. graevenitzii* infection (13).

The clinical symptoms of pulmonary actinomycosis are often non-specific, including fever, cough, dyspnea, or chest pain (24). Therefore, early diagnosis of the slowly progressing actinomycosis is difficult. In eight cases, patients were required be distinguished from tuberculosis, cancer, pulmonary coccidioidomycosis, or atypical pneumonia.

Radiological findings of pulmonary actinomycosis are also non-specific, including mass (23), nodules, patchy infiltrates, segmental air-space consolidation, and cavitation. It’s often confused with malignancy or tuberculosis. A characteristic CT finding is a central low density within the parenchymal consolidation and adjacent pleural thickening (25, 26). Initially, the disease is present as a small, poorly defined nodule in the peripheral lung, with or without an interlobular septal thickening. Gradually, the nodule develops into a segmental air-space consolidation or mass. With the slow progression of infection, a cavity forms in central areas of low attenuation. CT enhanced images may show rim-like peripheral enhancement and multiple central low-attenuation areas. Further progression of the disease may involve the pleura, the chest wall, or the neighboring pulmonary lobes (25–28). In the eight cases, four cases showed nodules, three cases showed air-space consolidation. And five of eight cases had a cavity formation.

Bacterial cultures and histopathological features of biopsy specimens are the cornerstone of diagnosis. However, it is challenging to confirm the presence of bacterial by culture due to antibiotic treatment, concomitant organisms growth, or inadequate conditions (1). Therefore, this requires clear communication of the suspicion of actinomycosis with the microbiology laboratory. With the development of techniques, more bacterial identification methods are increasingly used, including 16S rRNA gene sequence analysis, next-generation sequencing (29) or MALDI-TOF/MS. In our case, the patient was diagnosed by bronchoalveolar lavage fluid culture using mass spectrometry, with a consolidation with central low density on chest CT. In addition, PCR analysis and 16S rRNA gene sequencing analysis are used to identify pathogens in four cases (10–12, 15). For pathology, the main non-surgical diagnostic methods are ultrasound or CT-guided percutaneous lung biopsy and transbronchial biopsy, with a high positive rate. The identification of actinomycete hyphae and sulfur particles in biopsy samples is the gold standard for diagnosis. In the eight cases, pulmonary *A. graevenitzii* infection was confirmed *via* sulfur granules or Actinomyces species detected in sputum (10), bronchoalveolar lavage (BAL) (12, 13, 15, 16), video-assisted thoracoscopic lung biopsy (11, 12), or transbronchial needle aspiration biopsy of lymph node guided by endoscopic ultrasonography (14).

A long term beta-lactam antibiotic therapy is needed for patiens with pulmonary actinomycosis. The intravenous administration of penicillin G is recommended for 2 to 6 weeks, followed by oral administration of penicillin V or amoxicillin for 6 to 12 months (1). It may be necessary to perform surgical management to drain voluminous abscesses, marsupialize chronic sinus tracts, and excise fibrotic lesions. All the eight cases were treated with antibiotics, mainly beta-lactams, and the duration of treatment varied from 5 weeks to 7 months with one case not mentioned. Of note, our case was successfully treated for 7 months and was being followed up closely. Pleasingly, his clinical status and imaging presentations were getting better.

In conclusion, our case and literature review indicated that pulmonary *A. graevenitzii* infection was a rare disease with non-specific clinical characteristics. The diagnosis is mainly based on pathogen identification and histopathology. We need to consider the possibility of pulmonary actinomycosis when investigating a consolidation shadow with necrosis or *trans*-fissural extension. Bronchoscopy plays a key role in the non-invasive diagnostic procedure, providing specimens to make the final diagnosis. In addition, matrix- assisted laser desorption/ionization time-of-flight mass spectrometry is an accurate tool for *Actinomyces* identification.

## Ethics Statement

Written informed consent was obtained from the individual(s) for the publication of any potentially identifiable images or data included in this article.

## Author Contributions

YY and ZH: concept and writing of the manuscript. DP: interpretation of the sources and patient acquisition. ZX: image example. JW and SZ: supervision and concept. All authors read and approved the final draft, contributed to the article, and approved the submitted version.

## Conflict of Interest

The authors declare that the research was conducted in the absence of any commercial or financial relationships that could be construed as a potential conflict of interest.

## Publisher’s Note

All claims expressed in this article are solely those of the authors and do not necessarily represent those of their affiliated organizations, or those of the publisher, the editors and the reviewers. Any product that may be evaluated in this article, or claim that may be made by its manufacturer, is not guaranteed or endorsed by the publisher.
